# Pancreatic cancer: Cutaneous metastases, clinical descriptors and outcomes

**DOI:** 10.1002/cam4.4916

**Published:** 2022-06-06

**Authors:** Lilly Gu, Paras P. Mehta, Devika Rao, Veronica Rotemberg, Marinela Capanu, Joanne Chou, Sabrina Lin, Carlie S. Sigel, Klaus J. Busam, Lindsay Boyce, Allison Gordon, Eileen M. O'Reilly

**Affiliations:** ^1^ Memorial Sloan Kettering Cancer Center New York New York USA; ^2^ Weill Cornell Medicine New York New York USA; ^3^ Gastrointestinal Oncology Service, Department of Medicine Memorial Sloan Kettering Cancer Center New York New York USA; ^4^ Dermatology Service Memorial Sloan Kettering Cancer Center New York New York USA; ^5^ Department of Epidemiology & Biostatistics Memorial Sloan Kettering New York New York USA; ^6^ Department of Pathology Memorial Sloan Kettering Cancer Center New York New York USA; ^7^ Memorial Sloan Kettering Library Memorial Sloan Kettering Cancer Center New York New York USA; ^8^ David M. Rubenstein Center for Pancreatic Cancer, MSK New York New York USA

**Keywords:** cutaneous metastasis, pancreatic cancer, Sister Mary Joseph nodule, umbilical nodule

## Abstract

**Background:**

Cutaneous metastases in pancreatic cancer (PC) are rare. Herein, we evaluate the clinical, genomic, and other descriptors of patients with PC and cutaneous metastases.

**Methods:**

Institutional databases were queried, and clinical history, demographics, PC cutaneous metastasis details, and overall survival (OS) from cutaneous metastasis diagnosis were abstracted. OS was estimated using Kaplan–Meier methods.

**Results:**

Forty patients were identified, and median age (Q1–Q3, IQR) of PC diagnosis was 66.0 (59.3–72.3, 12.9) years. Most patients had Stage IV disease at diagnosis (*n* = 26, 65%). The most common location of the primary tumor was the tail of the pancreas (*n* = 17, 43%). The most common cutaneous metastasis site was the abdomen (*n* = 31, 78%), with umbilical lesions occurring in 74% (*n* = 23) of abdominal lesions. The median OS (95% CI) was 11.4 months (7.0, 20.4). Twenty‐three patients had umbilical metastases (58%), and 17 patients had non‐umbilical metastases (43%). The median OS (95% CI) was 13.7 (7.0, 28.7) months in patients with umbilical metastases and 8.9 (4.1, Not reached) months in patients with non‐umbilical metastases (*p* = 0.1). Sixteen of 40 (40%) patients underwent somatic testing, and findings were consistent with known profiles. Germline testing in 12 (30%) patients identified pathogenic variants in patients: *CHEK2*, *BRCA1,* and *ATM.*

**Conclusion:**

Cutaneous metastases from PC most frequently arise from a pancreas tail primary site and most frequently occur in the umbilicus. Cutaneous metastases may generally be categorized as umbilical or non‐umbilical metastases.

## Graphical abstract

Cutaneous Metastasis from Pancreatic Cancer. (A) pink, scaly, eroded papulonodule. (B) moderately to poorly differentiated adenocarcinoma in the dermis. (C) tumor cells are immunoreactive for cytokeratin 7.

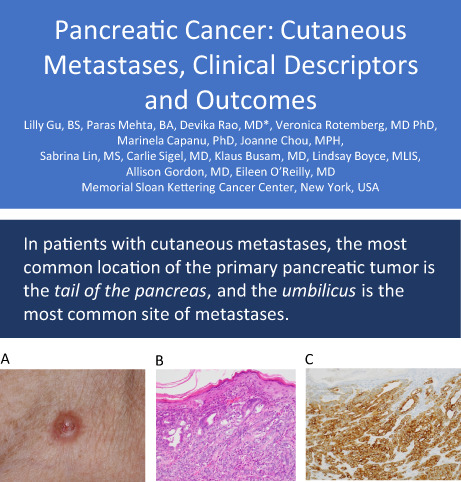

## 1 INTRODUCTION

Pancreatic cancer (PC) is the seventh leading cause of cancer‐related death in the world, with a 5‐year survival rate of 10%.^1^ A majority of patients present with metastatic disease at diagnosis of PC. The most frequent sites of metastasis are liver, lymph nodes, and peritoneum, followed by lungs, bones, and rarely brain and skin.^1^, ^2^

The occurrence of cutaneous metastasis from PC is rare with the exact incidence unknown. Cubilla and Fitzgerald reported nine out of 119 patients (7.6%) with PC exhibiting cutaneous metastasis at autopsy.^3^ Lookingbill et al. reported that of 420 cancer patients with cutaneous metastases, only two cases were identified to be of pancreatic origin (0.5%).^4^ Brownstein and Helwig found that of 724 patients with cutaneous metastasis, 2.0% were from PC.^5^ To place these numbers in the context of cutaneous metastases from other cancers, Lookingbill et al. noted for patients with cutaneous metastases, melanoma (18.3%), and breast cancer (50.5%) contribute to the majority of cases, and colorectal cancer accounts for about 4. 3% of cases with cutaneous metastases.^4^

Our understanding of the pathobiology of PC and cutaneous metastases is primarily based on single case descriptions in the literature.^6^, ^7^, ^8^, ^9^, ^10^, ^11^ The most common site of cutaneous metastasis of PC is the umbilicus, also referred to as ‘Sister Mary Joseph's nodule’.^12^ There is disagreement as to whether these lesions represent cutaneous or deeper‐seated peritoneal lesions that spread to the umbilicus.^13^ Other reported sites of cutaneous metastases include the face, neck, scalp, temple, chin, axilla, chest, abdomen, buttocks, scrotum, and labia.^7^, ^11^, ^14^, ^15^ Clinically, a cutaneous metastasis most commonly presents as a red nodule or mass but may also present as a plaque or swelling of the skin.^14^

Herein, we report on a large single‐institution cohort of patients with PC and cutaneous metastases and provide a detailed summary of clinical descriptors and outcomes and place these data in the context of the reported literature.

## 2 METHODS

### 2.1 Search of the EMR

Memorial Sloan Kettering (MSK) Institutional Review Board approval was obtained to conduct a DATALINE search of the MSK patient database using the search terms “pancreas cancer” and “cutaneous mets” based on International Classification of Disease (ICD) billing codes C25 (malignant neoplasm of pancreas) and C79.2 (secondary malignant neoplasm of skin). The search timeline was from 1/1/2000 to 2/4/2021. Patient charts were screened to exclude patients who did not have a history of PC and cutaneous or subcutaneous metastasis.

### 2.2 Chart review

Various parameters were collected, including demographics (age, gender, race, ethnicity), patient history (social history, past abdominal surgical history), PC details (date of diagnosis, location of primary tumor, histologic subtype, grade, stage, location of metastases at diagnosis, treatment, date of death, cutaneous metastases details (location, date noted clinically, physical exam findings, skin biopsy results, treatment), and genetic testing (somatic or germline mutations, microsite instability score, tumor mutation burden).

### 2.3 Statistical analysis

Baseline characteristics were compared by type of cutaneous metastases utilizing Fisher's exact tests. Overall survival (OS) was calculated from the cutaneous metastasis diagnosis date until the date of death or last follow‐up and estimated using the Kaplan–Meier method. Comparison of OS between non‐umbilical metastases vs umbilical metastases was done using the log‐rank test. The impact of local treatment on the OS of patients with cutaneous metastases was analyzed with a time‐dependent univariate Cox regression model,^16^ as the local treatment date cannot be considered as a baseline covariate due to its collection after follow‐up time begins. All statistical analyses were performed with R (version 4.1.0, R foundation). All *P*‐values were two‐sided. *p* < 0.05 were considered to indicate statistical significance.

### 2.4 Literature review

A literature search of the PubMed and Embase databases was conducted through 08/02/2021, using controlled vocabulary and keywords for pancreatic cancer, cancer metastasis, and cutaneous cancer. Abstracts were reviewed, and articles unrelated to PC and cutaneous metastases were eliminated. Full articles were then reviewed, and the following parameters were obtained: patient age at PC diagnosis, sex, stage at diagnosis, PC histologic subtype, primary PC location, months between cutaneous metastasis presentation, and prognosis. Reference lists of the articles were also screened to identify additional eligible articles. Non‐English articles were excluded from the study.

## 3 RESULTS

**FIGURE 1:**
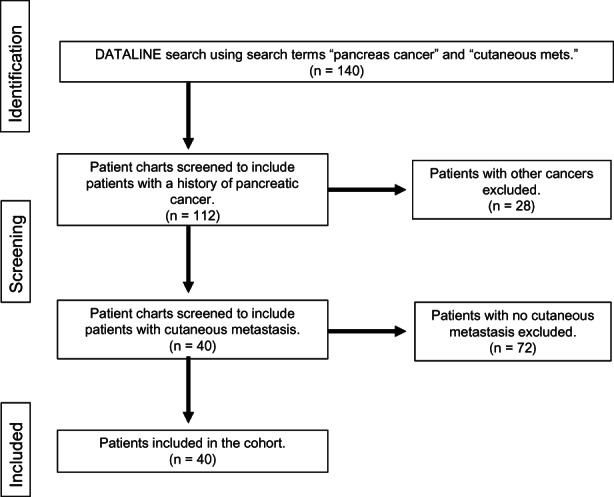
Cohort diagram.

For the patient cohort, the institutional search yielded 140 patients, and the final cohort for analysis comprised of *n* = 40 patients with PC and cutaneous metastasis (Figure 1).

### 3.1 Demographics

Demographic characteristics are summarized in Table 1 and Table S1. For the 40‐patient cohort, *n* = 21 (53%) were male and the majority were white, *n* = 32 (80%). The median age (Q1–Q3, IQR) of PC diagnosis was 66.0 (59.3–72.3, 12.9) years.

**TABLE 1:** Pancreatic cancer characteristics of *n* = 40 patients with cutaneous metastasis

Sex (*n* = 40)	*N*	Percentage
Male	21	53%
Female	19	48%
Race (*n* = 40)		
White	32	80%
Black	3	8%
Asian	2	5%
Unknown	3	8%
Age at diagnosis of pancreatic cancer (*n* = 40)		
<50 years	4	10%
50–65 years	16	40%
>65 years	20	50%
Presenting symptom^a^		
Abdominal pain	17	43%
Weight loss	8	20%
Fatigue	6	15%
Back pain	4	10%
Umbilical nodule or bleeding	6	15%
Jaundice	4	10%
Nausea	2	5%
Incidental	3	8%
Other	10	25%
Location of primary tumor (*n* = 40)		
Head	11	28%
Body	10	25%
Neck	1	3%
Tail	17	43%
Unclear	1	3%
Histologic subtype (*n* = 40)		
Adenocarcinoma	39	98%
Neuroendocrine	1	3%
Grade (*n* = 40)		
Well differentiated	2	5%
Moderately differentiated	16	40%
Poorly differentiated	15	38%
Unknown	7	18%
Stage at diagnosis (*n* = 40)		
I	2	5%
II	10	25%
III	2	5%
IV	26	65%
Therapy (*n* = 40)		
Surgery	14	35%
Chemotherapy	37	93%
Radiation	16	40%
Somatic sequencing (*n* = 16)		
*KRAS*	16	100%
G12D	7	44%
G12V	4	25%
G12R	2	13%
Other/Unknown	3	19%
*TP53*	7	44%
*CDKN2Ap14ARF*	5	31%
*CDKN2Ap16INK4A*	5	31%
*CDKN2B*	3	19%
Germline variants (*n* = 12)		
*CHEK2*	1	8%
*BRCA1*	1	8%
*ATM*	1	8%
None	9	75%

^**a**^ Presenting symptom is defined as the initial chief complaint that led to diagnosis of pancreatic cancer. Some patients had multiple presenting symptoms.

### 3.2 Pancreatic cancer characteristics

The most common presenting symptoms included abdominal pain (*n* = 17, 43%), weight loss (*n* = 8, 20%), fatigue (*n* = 6, 15%), and an umbilical nodule or bleeding (*n* = 6, 15%). Twenty patients (50%) had cutaneous metastases at diagnosis. Of the 20 patients, 19 patients (48%) had cutaneous metastases diagnosed within 30 days of initial presentation, and one patient was diagnosed with cutaneous metastases 1.2 months following PC diagnosis. The pancreatic tail was the most common location for the primary tumor (*n* = 17, 43%), followed by head (*n* = 11, 28%) and body (*n* = 10, 25%). The most common histology was adenocarcinoma (n = 39, 98%), and one patient had a neuroendocrine malignancy. The most common grades were moderately differentiated (*n* = 16, 40%) and poorly differentiated (*n* = 15, 38%). Most patients had Stage IV disease at diagnosis (*n* = 26, 65%). Thirty‐five percent of patients (*n* = 14) underwent surgical resection of primary tumor, and most patients received chemotherapy (*n* = 37, 93%), with one patient with neuroendocrine cancer receiving hormonal therapy and immunotherapy, and the remaining two patients died prior to initiating planned chemotherapy. Forty percent of patients (*n* = 16) received radiation therapy.

### 3.3 Cutaneous metastasis characteristics

**FIGURE 2:**
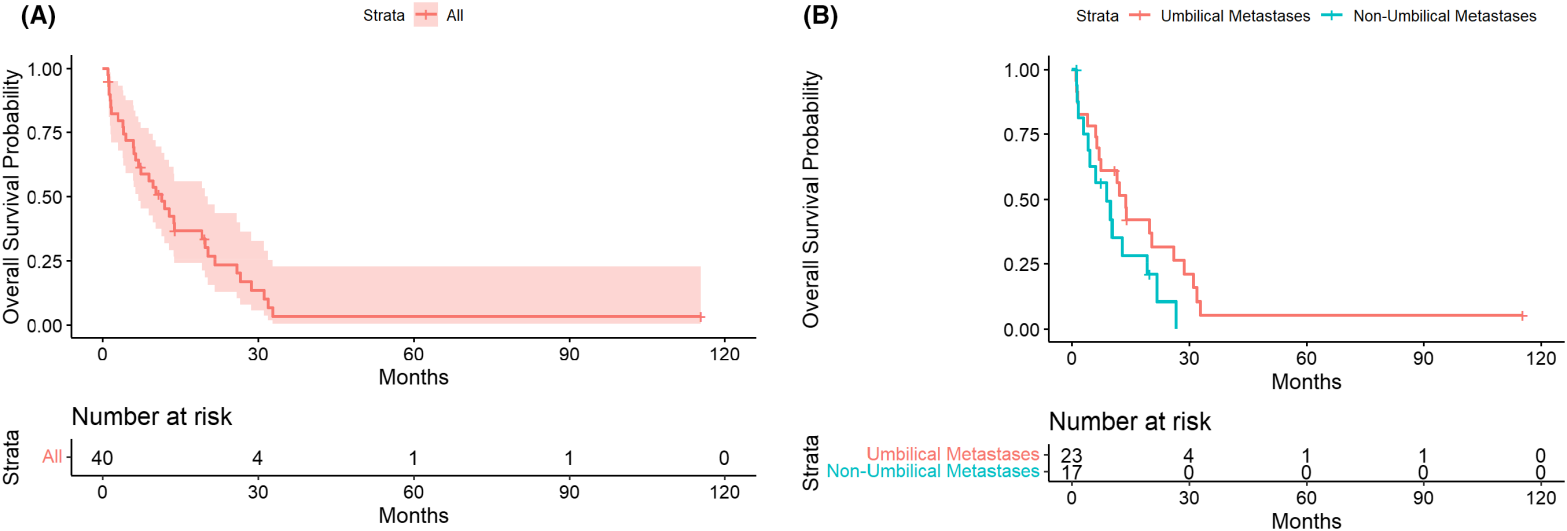
Overall survival curves from pancreatic cancer cutaneous metastases. (A) Overall survival of total cohort (*n* = 40). (B) Overall survival stratified by umbilical metastases and non‐umbilical metastases (*p* = 0.1).

**FIGURE 3:**
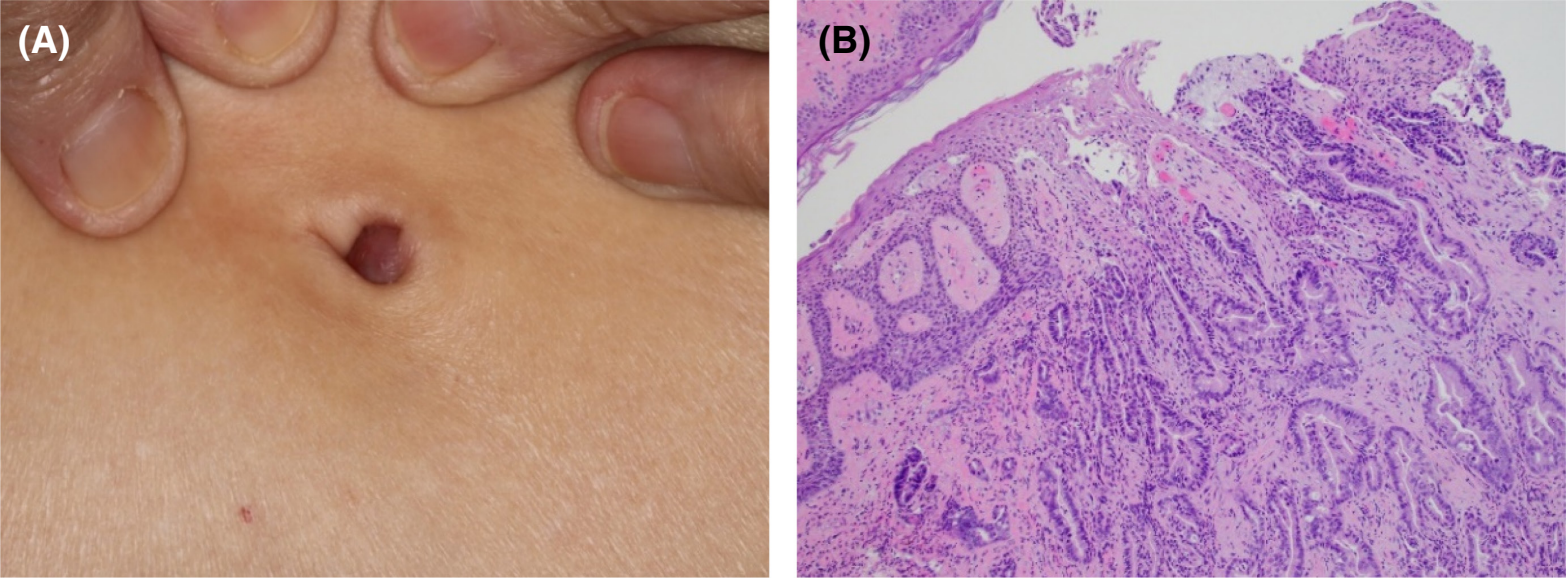
Cutaneous umbilical metastasis from pancreatic cancer. (A) Clinical appearance of a cutaneous umbilical metastasis, described as a violaceous to red umbilical papulonodule. (B) The microscopic findings of the skin biopsy show infiltration of the dermis by a ductal adenocarcinoma. Its microscopic features are consistent with a pancreatic origin.

**FIGURE 4:**
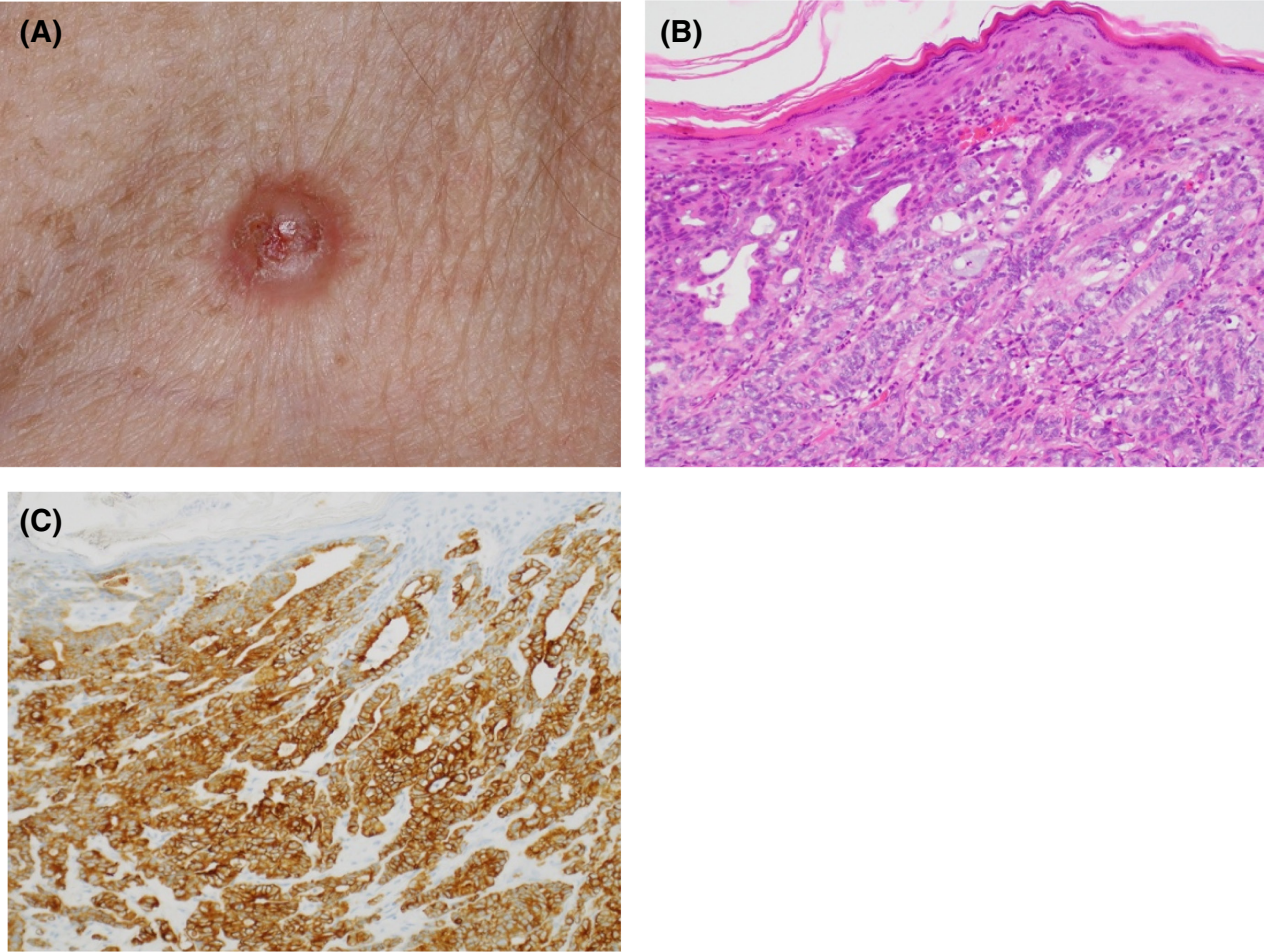
Cutaneous non‐umbilical metastasis from pancreatic cancer. (A) Clinical appearance of a cutaneous neck metastasis, described as a pink, scaly, eroded papulonodule. (B) Histopathologically, there is a moderately to poorly differentiated adenocarcinoma in the dermis. (C) The tumor cells are immunoreactive for cytokeratin 7.

A detailed summary of cutaneous metastasis characteristics is provided in Table 2 and Table S1. Among these 40 patients, the median interval (Q1–Q3, IQR) between diagnosis of PC and development of cutaneous metastasis was 1.4 (0–13.5, 13.5) months. The median OS (95% CI) from cutaneous metastasis diagnosis was 11.4 months (7.0, 20.4) (Figure 2). Eighteen patients (45%) had cutaneous metastases at more than one location. The most common cutaneous metastasis site was the abdomen (*n* = 31, 78%), with umbilical lesions occurring in 74% (*n* = 23) of abdominal lesions. Less commonly, cutaneous metastases occurred on the posterior thorax (*n* = 5, 13%) or scalp (*n* = 4, 10%). The most common physical exam finding was a nodule or a mass (*n* = 35, 88%). Photographs of a cutaneous umbilical metastasis and cutaneous neck metastasis with corresponding histology pictures are illustrated in Figures 3 and 4, respectively. Cutaneous metastases occurred most commonly in setting of the pancreatic tumor primary site located in the tail (*n* = 17, 43%), followed by head (*n* = 11, 28%) and body (*n* = 10, 25%).

**TABLE 2:** Summary of *n* = 40 patients with pancreatic cancer and cutaneous metastasis characteristics

Location	Count	Percentage
Umbilicus	23	58%
Other abdomen	17	43%
Pelvis^a^	1	3%
Scalp	4	10%
Face	2	5%
Neck	2	5%
Chest	1	3%
Breast	1	3%
Back	5	13%
Axilla	2	3
Upper extremity	1	5%
Lower extremity	2	3%
Onset of cutaneous metastasis relative to chemotherapy (*n* = 40)^b^		
Before	20	50%
During	11	28%
Break	9	23%
First‐line chemotherapy regimen^b^		
**Stage IV cancer**	**26**	**65%**
Before	20	
Gemcitabine based	12	60%
5‐FU based	5	25%
Other	1	5%
No chemotherapy	2	10%
During	6	
Gemcitabine based	1	17%
5‐FU based	5	83%
**Non‐metastatic cancer**	**14**	**35%**
During	5	
Gemcitabine based	3	60%
5‐FU based	1	20%
Other	1	20%
Break	9	
Gemcitabine based	7	78%
5‐FU based	1	11%
Other	1	11%
Number of chemotherapy lines before cutaneous metastasis (*n* = 40)		
0	20	50%
1	13	33%
2	3	8%
3	3	8%
4	1	3%
Number of chemotherapy lines after cutaneous metastasis (*n* = 40)		
0	6	15%
1	16	40%
2	8	20%
3	8	20%
4	2	5%
First‐line chemotherapy regimen for stage IV cancer (*n* = 24)		
Gemcitabine based	13	54%
5‐FU based	10	42%
Other	1	4%
First‐line chemotherapy regimen for non‐metastatic cancer (*n* = 14)		
Gemcitabine based	10	71%
5‐FU based	2	14%
Other	2	14%
Skin biopsy (*n* = 40)		
Yes	35	88%
No	5	13%
Treatment of cutaneous metastasis (*n* = 40)		
Excision	11	28%
Local radiation	1	3%
Excision and radiation	2	5%
None	26	65%
Time from development of pancreatic cancer and occurrence of cutaneous metastasis		
0–1 month	19	48%
1–6 months	4	10%
6–12 months	5	13%
>12 months	12	30%

^a^ Pelvis refers to iliopsoas subcutaneous nodule.

^b^ Before refers to diagnosis of cutaneous metastases before the initiation of chemotherapy. During refers to diagnosis of cutaneous metastases during chemotherapy. Break refers to diagnosis of cutaneous metastases during a chemotherapy break.

Twenty patients (50%) developed cutaneous metastases prior to initiation of chemotherapy. Eleven patients (28%) developed cutaneous metastases during chemotherapy, with the development of cutaneous metastasis occurring within 1 month of treatment for one patient (9%), between 1–6 months for three patients (27%), and over 6 months in seven patients (64%). Nine patients (23%) developed cutaneous metastases during a break from systemic therapy. Following development of cutaneous metastasis, most patients received one line of chemotherapy (*n* = 16, 40%) with a smaller number receiving two lines in *n* = 8 (20%) and three lines in *n* = 8 (20%). Of the patients presenting with Stage IV PC and treated with chemotherapy (*n* = 24), 13 patients (54%) received first‐line chemotherapy with a gemcitabine backbone, and 10 patients (42%) received first‐line chemotherapy with a fluorouracil (5‐FU) backbone. In contrast, among patients presenting with non‐metastatic cancer (*n* = 14), the majority received adjuvant chemotherapy with gemcitabine (*n* = 10, 71%).

Of those presenting with Stage IV cancer (*n* = 26), six patients (23%) developed cutaneous metastases while receiving chemotherapy. Whereas among those presenting with non‐metastatic cancer (*n* = 14), five patients (36%) developed cutaneous metastases while on chemotherapy, and nine patients (64%) of patients developed cutaneous metastases while on a treatment break.

Following development of cutaneous metastasis, 11 patients underwent local excision (28%), one patient received radiation (3%), and two patients received both local excision and radiation (5%). Patients with cutaneous metastases who received local treatment were 16% less at risk to die compared to patients who did not receive local treatment, with a hazard ratio (CI) of 0.84 (0.11, 6.14); however, this difference was not statistically significant (*p* = 0.86).

Twenty‐three patients had umbilical metastases (58%), and 17 patients had non‐umbilical metastases (43%). A higher proportion of patients with umbilical metastases (*n* = 17, 74%) presented with Stage IV disease, in comparison to patients who had non‐umbilical metastases (*n* = 9, 53%), but this difference was not significant (*p* = 0.2). A significantly higher proportion of patients with umbilical metastases had cutaneous metastases at PC diagnosis (*n* = 15, 65%) in comparison to those with non‐umbilical cutaneous metastases (*n* = 4, 24%) (*p* = 0.01). A slightly higher proportion of patients with umbilical metastases had primary tumors from the body or tail versus head or neck of the pancreas (*n* = 17, 74%) in comparison to patients with non‐umbilical cutaneous metastases (*n* = 10, 62%), but this difference was not significant (*p* = 0.4). Eighty‐three percent (*n* = 19) of patients with umbilical metastases vs. 59% (*n* = 10) of patients with non‐umbilical metastases also had peritoneal metastases. Twenty‐five patients had ascites. Seventy‐two percent (*n* = 21) of patients with peritoneal metastases had ascites, versus 36% of patients without peritoneal metastases. The median OS (95% CI) was 13.7 (7.0, 28.7) months in patients with umbilical metastases and 8.9 (4.1, Not reached) months in patients with non‐umbilical metastases (*p* = 0.1) (Figure 2).

### 3.4 Somatic and germline testing

The results of MSK IMPACT genetic testing ^17^ are summarized in Table 1. Sixteen of 40 (40%) patients underwent somatic testing. The most frequently mutated genes were, *KRAS* (*n* = 16, 100%), *TP53* (*n* = 7, 44%), *CDKN2Ap14ARF* (*n* = 5, 31%), *CDKN2Ap16INK4A* (*n* = 5, 31%), and *CDKN2B* (*n* = 3, 19%). Germline testing was undertaken in 12 (30%) patients and pathogenic or likely pathogenic variants were observed in three patients: *CHEK2* (*n* = 1, 8%), *BRCA1* (*n* = 1, 8%) and *ATM* (*n* = 1, 8%).

### 3.5 Literature review

The initial PubMed search yielded 24 articles. Reference lists of the articles were also screened for additional eligible articles, resulting in the inclusion of a total of 74 articles, comprising 90 cases of cutaneous metastases of PC. Table S2 summarizes the published literature. The median age (*n* = 88) at diagnosis of PC (Q1–Q3, IQR) was 65 (58–74, 17) years. The cases consisted of 45 males, 43 females, and two unknown. Stage at diagnosis was reported in 56 patients, with 51 patients diagnosed at Stage IV. Of the 73 patients with reported subtype, 68 patients had adenocarcinoma, and five patients had neuroendocrine tumors. Forty‐eight patients had cutaneous metastases present at PC diagnosis, and 18 patients did not; presence of cutaneous metastases at diagnosis of PC was unknown in 24 of patients. The location of cutaneous metastases was reported 89 patients; 39 patients had umbilical metastases, and 50 patients had non‐umbilical cutaneous metastases. The primary site of pancreatic cancer was the pancreatic body or tail in 54 patients, and the head in 18 patients; location of primary tumor was not reported in 18 patients. For patients with umbilical metastases (*n* = 39), 32 had primary tumor of the pancreas tail or body, 0 had primary tumor of the pancreas head, and seven were unreported. For patients with non‐umbilical cutaneous metastases (*n* = 50), 22 had primary tumor of the pancreas tail or body, 17 had primary tumor of the pancreas head, 11 were unreported.

## 4 DISCUSSION

Cutaneous metastases from PC represent a rare entity which has not been well studied and the prior literature comprises mostly of single case reports. We conducted a detailed clinical and pathobiological analysis of a cohort of 40 patients with cutaneous metastases from PC. From our study, we observed that the umbilicus was the most common site of cutaneous metastases, potentially attributed to its rich vascularization, intersection of lymphatic channels, proximity to the peritoneum, and connections to embryological remnants. ^18^ Differences between patients with and without peritoneal dissemination should be explored in future studies. The clinical course of cutaneous metastasis can be quite variable. Fifty percent of patients (*n* = 20) presented with cutaneous metastasis at diagnosis of PC, but almost half of the cohort (*n* = 17, 43%) developed cutaneous metastasis more than 6 months following initial diagnosis of PC. Cutaneous metastases can generally be classified into umbilical metastases and non‐umbilical metastases. In our study, we observed that slightly higher, but nonsignificant, proportion of patients with umbilical metastases had a pancreas tail or body primary (compared to head) versus non‐umbilical metastases (74% vs. 62%, *p* = 0.4), and a similar finding was observed with published cases in the literature (82% vs. 44%). Patients with umbilical metastases did not have a higher frequency of metastasis (Stage IV diagnosis) at initial diagnosis of PC in comparison to those with non‐umbilical cutaneous metastases (*p* = 0.2), but patients with umbilical metastases did have a significantly higher frequency of cutaneous metastases at PC diagnosis in comparison to those with non‐umbilical metastases, possibly suggesting a different mechanism of cancer spread (*p* = 0.01). Median overall survival for patients was umbilical metastases (14 months) was higher than the patients with non‐umbilical metastases (8.9 months), although this difference was not statistically significant (*p* = 0.1).

Cutaneous metastases from PC have been reported in multiple case reports in the literature.^6^, ^7^, ^8^, ^9^, ^10^, ^11^ Horino et al. reviewed 42 cases of pancreatic metastasis in the literature from 1950 to 2011, with the majority of patients presenting with umbilical cutaneous metastases and presenting predominantly with subcutaneous nodules,^19^ consistent with the results from our study. Horino also noted that the incidence of umbilical metastases from cancers of the pancreatic body and tail was significantly more frequent than from the pancreatic head. ^19^ Our study found that although cutaneous metastases from the pancreatic body and/or tail accounted for a slightly higher proportion of umbilical versus non‐umbilical cutaneous metastases, this difference was not significant. For the patients between 1950 and 2011, Horino et al. observed that the median OS following diagnosis of cutaneous metastasis was 5.1 months,^19^ and the median OS (95% CI) in our 40‐patient cohort was 11.4 months (7.0, 20.4), likely reflective of survival improvements in systemic therapy during the last two decades.

Previous studies have reported cutaneous metastases to be an initial manifestation of PC in a variable proportion of cases, over 90% in some cases^19^, ^20^ and 55.6% in another report.^21^ In our study, 50% of patients had cutaneous metastasis at presentation with pancreas cancer. In our review of the literature, eight of 59 patients with abdominal metastases (13.6%) had abdominal metastases in association with a surgical procedure, including fine‐needle tract, drains, or abdominal surgery. From our study, one out of 31 (3.2%) patients with abdominal metastases had abdominal metastases in association with a surgical procedure. This may represent an important unintended mechanism of spread.

For the cohort who underwent somatic testing, the observed mutational spectrum and frequency, *KRAS*, *TP53*, and *CDKN2A*, were consistent with the published literature.^22^

While skin lesions were biopsied in most patients to confirm the diagnosis, cutaneous metastases were rarely treated with locoregional therapy, and most patients were treated with systemic therapy. However, in the small proportion of patients who received locoregional therapy, there did seem to be a modest benefit, although this is not significantly associated with survival status. In all of the cases, the treating physician did not recommend local treatment or removal of cutaneous metastases in patients with widespread metastases, likely due to the anticipated lack of impact on cancer outcome in the setting of disseminated disease. In settings where patients had cutaneous metastases removed or treated with radiation (*n* = 14), these interventions occurred in the setting of symptomatic disease and/or patient preference. Given the retrospective nature of the study, we are unable to comment on the impact cutaneous metastases had on the quality of life of patients; however, this is an important area of study for future research.

Limitations of this study include the retrospective nature of the study design, single‐institution cohort and limited ethnic and racial diversity. There is a possibility some patients with PC and cutaneous metastasis were inadvertently omitted. In cases of cutaneous umbilical metastases, differentiating between true cutaneous metastases vs. peritoneal metastases is challenging, as skin involvement is not commented on in the majority of the biopsies. In addition, longitudinal changes in cutaneous metastasis over time were not well described in the medical record, thus therapeutic impacts from systemic therapy on cutaneous metastases were difficult to ascertain. Three patients were lost to follow‐up, and only a small fraction of the cohort had undergone genetic testing, limiting conclusions that can be drawn.

In conclusion, cutaneous metastases from PC are rare and can be present at the time of diagnosis of stage IV disease. The most common location of the primary tumor is the tail of the pancreas, and the umbilicus is the most common site of cutaneous metastasis. In comparison to non‐umbilical cutaneous metastases, umbilical metastases are more likely to be associated with cutaneous metastases at pancreatic cancer diagnosis (*p* = 0.01) and have a slightly better prognosis (*p* = 0.1), which may be underpinned by different disease biology.

## AUTHOR CONTRIBUTIONS

Lilly Gu: data curation, formal analysis, writing ‐ original draft, writing ‐ review and editing. Paras P. Mehta: data curation, writing ‐ review and editing. Devika Rao: conceptualization, formal analysis, project administration, supervision, writing ‐ review and editing. Veronica Rotemberg: conceptualization, writing ‐ review and editing. Marinela Capanu: formal analysis, writing ‐ review and editing. Joanne Chou: formal analysis, writing ‐ review and editing. Sabrina Lin: formal analysis, writing ‐ review and editing. Carlie S. Sigel: resources, writing ‐ review and editing. Klaus J. Busam: resources, writing ‐ review and editing. Lindsay Boyce: resources, writing ‐ review and editing. Allison Gordon: conceptualization, resources, writing ‐ review and editing. Eileen M. O′ Reilly: conceptualization, formal analysis, funding acquisition, project administration, supervision, writing ‐ review and editing.

## FUNDING INFORMATION

Cancer Center Support Grant/Core Grant P30 CA008748.

## CONFLICT OF INTEREST

LG: None; PM: None; DR: None; VR: : Inhabit Brands, Inc.; MC: None; JC: None; SL: None; CS: None; KB: : Dermtech; : Elsevier, Springer; LB: None; AG: None; EMO'R: : Genentech/Roche, Celgene/BMS, BioNTech, BioAtla, AstraZeneca, Arcus, Elicio, Parker Institute, AstraZeneca; : Cytomx Therapeutics (DSMB), Rafael Therapeutics (DSMB), Silenseed, Tyme, Seagen, Molecular Templates, Boehringer Ingelheim, BioNTech, Ipsen, Polaris, Merck, IDEAYA, AstraZeneca, Noxxon, BioSapien, Cend Therapeutics, Thetis, Bayer (spouse), Genentech‐Roche (spouse), Celgene‐BMS (spouse), Eisai (spouse).

## ETHICS STATEMENT

IRB approval: Reviewed and approved by Memorial Sloan Kettering Cancer Center. Review Board, Protocol # 20–351.

## PRECIS

This retrospective study evaluates a 40‐patient cohort with pancreatic cancer and cutaneous metastases, representing the largest series to date. Key findings are that the most common location of the primary tumor was the tail of the pancreas, and the umbilicus is the most common site of cutaneous metastasis. In comparison to non‐umbilical cutaneous metastases, umbilical metastases are more likely to be associated with cutaneous metastases at pancreatic cancer diagnosis (*p* = 0.01), which may be underpinned by a different disease biology.

## Supporting information


Supplementary Table 1

Supplementary Table 2

## Data Availability

The data that support the findings of this study are available from the corresponding author upon reasonable request.
