# Supplementary material for: Pancreatic cancer: Cutaneous metastases, clinical descriptors and outcomes
Source: Cancer Med. 2022 Jun 6;12(1):179–88. doi: 10.1002/cam4.4916 (PMC9844595; doi:10.1002/cam4.4916)
Supplement: Supplementary file 1 — Supplementary Table 1 [file CAM4-12-179-s002.docx]

|  | **Supplementary Table 1. Characteristics of n=40 Patient Cohort with Cutaneous Metastasis** | | | | | | | |  |
| --- | --- | --- | --- | --- | --- | --- | --- | --- | --- |
| Age at Diagnosis (years) | Sex | Decade of Diagnosis | Stage at Diagnosis | Primary Pancreas Tumor Site | Cutaneous Metastasis Location^a^ | Cutaneous Metastasis at Diagnosis | Time between Development of Pancreatic Cancer and Occurrence of Cutaneous Metastasis | Survival (months) from Diagnosis of Cutaneous Metastasis^b^ | |
| 67 | M | 1990s | I | head | Abdomen, umbilicus | N | 16.2 | 32.0 | |
| 52 | F | 2010s | I | head | Back | N | 28.1 | 6.0 | |
| 71 | M | 2000s | II | head | Abdomen, umbilicus | N | 9.6 | 7.0 | |
| 78 | M | 2010s | II | body | Abdomen, umbilicus | N | 14.7 | 6.0 | |
| 68 | M | 2000s | II | body | Abdomen | N | 14.9 | 8.9 | |
| 71 | M | 2010s | II | tail | Abdomen | N | 8.8 | 26.6 | |
| 79 | F | 2010s | II | tail | Umbilicus | N | 45.7 | 28.7 | |
| 67 | F | 2010s | II | head | Abdomen, umbilicus | N | 18.0 | 26.0 | |
| 52 | F | 2010s | II | head | Abdomen | N | 4.2 | - | |
| 66 | M | 2010s | II | head | Pelvis | N | 6.7 | 4.6 | |
| 38 | M | 2010s | II | head | Chest, umbilicus | N | 35.3 | 31.1 | |
| 65 | M | 2010s | II | tail | Scalp | N | 18.6 | 1.2 | |
| 77 | F | 2000s | III | head | Abdomen | N | 7.0 | 4.1 | |
| 48 | F | 2000s | III | head | Neck | N | 18.6 | 1.2 | |
| 59 | M | 2000s | IV | tail | Back, abdomen | N | 1.6 | 9.8 | |
| 47 | M | 2000s | IV | body | Umbilicus | Y | 0.0 | 1.2 | |
| 63 | M | 2000s | IV | body | Umbilicus | Y | 0.0 | 20.4 | |
| 50 | M | 2000s | IV | tail | Scalp, neck | Y | 0.9 | 19.2 | |
| 76 | F | 2000s | IV | tail | Abdomen, umbilicus | Y | 0.8 | 13.8 | |
| 52 | M | 2000s | IV | tail | Umbilicus | Y^c^ | 0.0 | 7.5 | |
| 74 | F | 2010s | IV | body | Abdomen, umbilicus | Y^c^ | 0.0 | 11.4 | |
| 68 | M | 2010s | IV | neck | Umbilicus, axilla | Y | 0.0 | 1.4 | |
| 59 | F | 2010s | IV | tail | Breast, face, lower extremity | Y | 0.6 | 10.3 | |
| 59 | F | 2010s | IV | tail | Umbilicus | N | 1.2 | - | |
| 46 | F | 2010s | IV | body | Umbilicus | Y^c^ | 0.0 | 32.8 | |
| 77 | F | 2010s | IV | tail | Umbilicus | Y^c^ | 0.0 | 4.0 | |
| 70 | M | 2010s | IV | tail | Abdomen, umbilicus | Y^c^ | 0.0 | 12.0 | |
| 63 | F | 2010s | IV | tail | Back, umbilicus | N | 14.4 | 1.7 | |
| 83 | F | 2010s | IV | body | Abdomen | Y | 0.0 | 21.8 | |
| 67 | F | 2010s | IV | body | Umbilicus | Y | 0.0 | 19.8 | |
| 73 | F | 2020s | IV | body | Abdomen, umbilicus | Y | 0.0 | 14.0, alive | |
| 63 | M | 2000s | IV | body | Abdomen | N | 13.2 | 12.8 | |
| 82 | F | 2000s | IV | head | Umbilicus | Y^c^ | 0.0 | 6.4 | |
| 50 | M | 2010s | IV | unknown | Upper extremity, face, scalp, back | Y | 0.0 | 3.0 | |
| 63 | F | 2010s | IV | tail | Axilla, lower extremity | N | 13.2 | 1.6 | |
| 73 | M | 2010s | IV | tail | Back, abdomen | N | 22.0 | 8.7, alive | |
| 59 | M | 2020s | IV | head | Scalp | N | 5.0 | - | |
| 70 | M | 2020s | IV | tail | Umbilicus | Y | 0.0 | 11.4, alive | |
| 59 | F | 2020s | IV | tail | Abdomen, umbilicus | Y | 0.9 | 1.0 | |
| 59 | M | 2010s | IV | tail | Umbilicus | Y | 0.0 | 13.7 | |

^a^Abdomen refers to non-umbilical cutaneous sites on the abdomen. ^b^Cutaneous metastasis at diagnosis is defined as presence of cutaneous metastasis before or within one month of pancreatic cancer diagnosis. ^c^These patients developed cutaneous metastases before formal pancreatic cancer diagnosis. - data is unknown, as patient was lost to follow-up.
