# Supplementary material for: Pancreatic cancer: Cutaneous metastases, clinical descriptors and outcomes
Source: Cancer Med. 2022 Jun 6;12(1):179–88. doi: 10.1002/cam4.4916 (PMC9844595; doi:10.1002/cam4.4916)
Supplement: Supplementary file 2 — Supplementary Table 2 [file CAM4-12-179-s001.docx]

| **Supplementary Table 2. Literature Summary of Pancreas Cancer and Cutaneous Metastases** | | | | | | | | | | |
| --- | --- | --- | --- | --- | --- | --- | --- | --- | --- | --- |
| Author | Year | Age | Sex | Stage | Pancreatic Cancer Site | Histologic Subtype | Cutaneous Metastasis Site | Cutaneous Metastasis at Diagnosis | Time from Development of Pancreatic Cancer and Occurrence of Cutaneous Metastasis | Survival (months) from Diagnosis of Cutaneous Metastasis |
| Edelstein[^1^](https://sciwheel.com/work/citation?ids=11698897&pre=&suf=&sa=0&dbf=0) | 1950 | 60 | M | IV | Body, tail | Adenocarcinoma | Face, neck | Y | 0.0 | 2.5 |
| Horn et al.[^2^](https://sciwheel.com/work/citation?ids=11698899&pre=&suf=&sa=0&dbf=0) | 1964 | 59 | F | - | - | - | Umbilicus | - | - | - |
| Horn et al.[^2^](https://sciwheel.com/work/citation?ids=11698899&pre=&suf=&sa=0&dbf=0) | 1964 | 50 | F | - | - | - | Umbilicus | - | - | - |
| Barrow et al.[^3^](https://sciwheel.com/work/citation?ids=11698900&pre=&suf=&sa=0&dbf=0) | 1966 | 65 | F | - | - | - | Umbilicus | - | - | - |
| Barrow et al.[^3^](https://sciwheel.com/work/citation?ids=11698900&pre=&suf=&sa=0&dbf=0) | 1966 | 44 | M | - | - | - | Umbilicus | - | - | - |
| Barrow et al.[^3^](https://sciwheel.com/work/citation?ids=11698900&pre=&suf=&sa=0&dbf=0) | 1966 | 57 | F | - | - | - | Umbilicus | - | - | - |
| Colin et al.[^4^](https://sciwheel.com/work/citation?ids=11698901&pre=&suf=&sa=0&dbf=0) | 1969 | 73 | F | IV | Head | Neuroendocrine | Abdomen | - | 12.0 | 9.0 |
| ^a^Sakai et al.[^5^](https://sciwheel.com/work/citation?ids=11698903&pre=&suf=&sa=0&dbf=0) | 1969 | 47 | M | - | Head | - | - | - | - | - |
| Bordin et al.[^6^](https://sciwheel.com/work/citation?ids=11698904&pre=&suf=&sa=0&dbf=0) | 1972 | 72 | M | IV | - | Adenocarcinoma | Umbilicus | Y | 0.0 | 3.2 |
| ^b^Charoenkul et al.[^7^](https://sciwheel.com/work/citation?ids=11698905&pre=&suf=&sa=0&dbf=0) | 1977 | 61 | M | - | Body | - | Umbilicus | - | - | - |
| Chakraborty et al.[^8^](https://sciwheel.com/work/citation?ids=11698906&pre=&suf=&sa=0&dbf=0) | 1977 | 62 | M | IV | Body | Adenocarcinoma | Upper extremity, umbilicus, abdomen | Y | 0.0 | - |
| Weiland et al.[^9^](https://sciwheel.com/work/citation?ids=11698907&pre=&suf=&sa=0&dbf=0) | 1978 | 76 | M | IV | - | Adenocarcinoma | Umbilicus | Y | 0.0 | - |
| Scarpa et al.[^10^](https://sciwheel.com/work/citation?ids=11698909&pre=&suf=&sa=0&dbf=0) | 1979 | 62 | F | IV | Tail | Adenocarcinoma | Umbilicus | Y | 0.0 | 12.0 |
| Scarpa et al.[^10^](https://sciwheel.com/work/citation?ids=11698909&pre=&suf=&sa=0&dbf=0) | 1979 | 36 | M | IV | Tail | Adenocarcinoma | Umbilicus | Y | 0.0 | 5.0 |
| Ferrucci et al.[^11^](https://sciwheel.com/work/citation?ids=11698911&pre=&suf=&sa=0&dbf=0) | 1979 | 75 | M | - | Body, tail | Adenocarcinoma | Abdomen^d^ | N | 3.0 | - |
| Smith et al.[^12^](https://sciwheel.com/work/citation?ids=11698912&pre=&suf=&sa=0&dbf=0) | 1980 | 54 | F | - | Body, tail | Adenocarcinoma | Abdomen^d^ | N | 3.0 | - |
| Chatterjee et al.[^13^](https://sciwheel.com/work/citation?ids=11698915&pre=&suf=&sa=0&dbf=0) | 1980 | 67 | M | IV | Tail | Adenocarcinoma | Umbilicus | Y | 0.0 | 2.0 |
| Shvili et al.[^14^](https://sciwheel.com/work/citation?ids=11698917&pre=&suf=&sa=0&dbf=0) | 1983 | 77 | M | IV | Tail | Adenocarcinoma | Umbilicus | Y | 0.0 | 4.0 |
| Frohlich et al.[^15^](https://sciwheel.com/work/citation?ids=11698918&pre=&suf=&sa=0&dbf=0) | 1986 | 75 | M | - | Tail | Adenocarcinoma | Abdomen^d^ | N | 2.0 | 1.0 |
| Rashleigh-Belcher et al.[^16^](https://sciwheel.com/work/citation?ids=11698920&pre=&suf=&sa=0&dbf=0) | 1986 | 68 | F | - | Head | Adenocarcinoma | Abdomen^d^ | N | 6.0 | - |
| ^c^Hisamoto et al.[^17^](https://sciwheel.com/work/citation?ids=11698926&pre=&suf=&sa=0&dbf=0) | 1987 | 76 | F | - | Tail | Adenocarcinoma | Umbilicus | - | - | 8.0 |
| Bergenfeldt et al.[^18^](https://sciwheel.com/work/citation?ids=11698929&pre=&suf=&sa=0&dbf=0) | 1988 | 60 | F | - | Head | Adenocarcinoma | Abdomen^d^ | N | 2.0 | 6.0 |
| Sironi et al.[^19^](https://sciwheel.com/work/citation?ids=11698930&pre=&suf=&sa=0&dbf=0) | 1991 | 72 | M | IV | Head | Adenocarcinoma | Lower extremity | N | 9.0 | 51.0, alive |
| Lookingbill et al.[^20^](https://sciwheel.com/work/citation?ids=11698867&pre=&suf=&sa=0&dbf=0) | 1993 | - | - | IV | - | Adenocarcinoma | Abdomen, chest, upper extremity | Y | 0.0 | 6.0 |
| Lookingbill et al.[^20^](https://sciwheel.com/work/citation?ids=11698867&pre=&suf=&sa=0&dbf=0) | 1993 | - | - | - | - | Adenocarcinoma | ^*^ | - | - | 6.0 |
| Siriwardena et al.[^21^](https://sciwheel.com/work/citation?ids=11698931&pre=&suf=&sa=0&dbf=0) | 1993 | 71 | M | IV | Body | Adenocarcinoma | Abdomen^d^ | Y | 0.0 | 2.0 |
| Taniguchi et al.[^22^](https://sciwheel.com/work/citation?ids=11698880&pre=&suf=&sa=0&dbf=0) | 1994 | 63 | F | IV | Head | Adenocarcinoma | Upper extremity, chest | Y | 0.0 | 27.0, alive |
| ^a^Ohashi et al.[^23^](https://sciwheel.com/work/citation?ids=11698983&pre=&suf=&sa=0&dbf=0) | 1995 | 79 | M | - | - | Adenocarcinoma | Neck, chest, abdomen | - | - | - |
| ^a^Ohashi et al.[^23^](https://sciwheel.com/work/citation?ids=11698983&pre=&suf=&sa=0&dbf=0) | 1995 | 65 | M | - | - | Adenocarcinoma | Back | - | - | - |
| ^a^Fukui et al. [^24^](https://sciwheel.com/work/citation?ids=12096255&pre=&suf=&sa=0&dbf=0) | 1995 | 49 | M | - | - | - | Face, chest | - | - | - |
| Puri et al.[^25^](https://sciwheel.com/work/citation?ids=11698939&pre=&suf=&sa=0&dbf=0) | 1995 | 45 | M | IV | - | Adenocarcinoma | Scalp, face, neck, back | Y | 0.0 | - |
| Nakano et al.[^26^](https://sciwheel.com/work/citation?ids=11698940&pre=&suf=&sa=0&dbf=0) | 1996 | 80 | M | IV | Tail | Adenocarcinoma | Scalp, face, upper extremity, chest, lower extremity | Y | 0.0 | 5.0 |
| Nakano et al.[^26^](https://sciwheel.com/work/citation?ids=11698940&pre=&suf=&sa=0&dbf=0) | 1996 | 80 | M | IV | Tail | Adenocarcinoma | Scalp | Y | 0.0 | 7.0 |
| Miyahara et al.[^27^](https://sciwheel.com/work/citation?ids=11698893&pre=&suf=&sa=0&dbf=0) | 1996 | 53 | M | IV | Body, tail | Adenocarcinoma | Umbilicus | Y | 0.0 | 5.0 |
| Miyahara et al.[^27^](https://sciwheel.com/work/citation?ids=11698893&pre=&suf=&sa=0&dbf=0) | 1996 | 76 | F | IV | Tail | Adenocarcinoma | Umbilicus | Y | 0.0 | 7.0 |
| Miyahara et al.[^27^](https://sciwheel.com/work/citation?ids=11698893&pre=&suf=&sa=0&dbf=0) | 1996 | 63 | M | IV | Tail | Adenocarcinoma | Umbilicus | Y | 0.0 | 4.0 |
| Miyahara et al.[^27^](https://sciwheel.com/work/citation?ids=11698893&pre=&suf=&sa=0&dbf=0) | 1996 | 65 | M | IV | Head | Adenocarcinoma | Face | Y | 0.0 | 4.0 |
| Miyahara et al.[^27^](https://sciwheel.com/work/citation?ids=11698893&pre=&suf=&sa=0&dbf=0) | 1996 | 43 | M | - | Head | Adenocarcinoma | Scalp | N | 15.0 | 3.0, alive |
| ^c^Lesur et al.[^28^](https://sciwheel.com/work/citation?ids=11698941&pre=&suf=&sa=0&dbf=0) | 1997 | 78 | M | - | Tail | Neuroendocrine | Umbilicus | - | - | 4.0 |
| Horino et al.[^29^](https://sciwheel.com/work/citation?ids=11698942&pre=&suf=&sa=0&dbf=0) | 1999 | 65 | F | I | Head | Adenocarcinoma | Chest | N | 16.0 | 8.0 |
| Florez et al.[^30^](https://sciwheel.com/work/citation?ids=11698944&pre=&suf=&sa=0&dbf=0) | 2000 | 48 | M | IV | Head | Adenocarcinoma | Buttock | Y | 0.0 | 10.0, alive |
| ^c^Kamata et al.[^31^](https://sciwheel.com/work/citation?ids=11698946&pre=&suf=&sa=0&dbf=0) | 2000 | 75 | F | - | Body | - | Umbilicus | - | - | 1.0 |
| Gawrieh et al.[^32^](https://sciwheel.com/work/citation?ids=11698947&pre=&suf=&sa=0&dbf=0) | 2002 | 45 | F | IV | Tail | Adenocarcinoma | Scalp | Y | 0.0 | 2.5 |
| Takeuchi et al.[^33^](https://sciwheel.com/work/citation?ids=11698948&pre=&suf=&sa=0&dbf=0) | 2003 | 77 | M | IV | Tail | Adenocarcinoma | Upper extremity | Y | 0.0 | - |
| Zhang et al.[^34^](https://sciwheel.com/work/citation?ids=11698950&pre=&suf=&sa=0&dbf=0) | 2003 | 34 | F | - | Body | Neuroendocrine | Umbilicus | N | 26.0 | - |
| ^c^Yoneda et al. [^35^](https://sciwheel.com/work/citation?ids=12096270&pre=&suf=&sa=0&dbf=0) | 2003 | 60 | F | - | Tail | - | Umbilicus | - | - | 2.0 |
| ^c^Yoneda et al.[^35^](https://sciwheel.com/work/citation?ids=12096270&pre=&suf=&sa=0&dbf=0) | 2003 | 53 | F | - | Tail | - | Umbilicus | - | - | 7.0 |
| Fiori et al.[^36^](https://sciwheel.com/work/citation?ids=11698971&pre=&suf=&sa=0&dbf=0) | 2003 | 65 | F | III | Head | Adenocarcinoma | Abdomen^d^ | N | 6.0 | 8.0, alive |
| St Peter et al.[^37^](https://sciwheel.com/work/citation?ids=11698972&pre=&suf=&sa=0&dbf=0) | 2003 | 75 | F | III | Head | Adenocarcinoma | Abdomen^d^ | N | 12.0 | 3.0 |
| Crescentini et al.[^38^](https://sciwheel.com/work/citation?ids=11698973&pre=&suf=&sa=0&dbf=0) | 2004 | 64 | F | IV | Body | Adenocarcinoma | Umbilicus | Y | 0.0 | 8.0, alive |
| ^c^Okazaki et al.[^39^](https://sciwheel.com/work/citation?ids=12096273&pre=&suf=&sa=0&dbf=0) | 2004 | 75 | M | - | Body | - | Umbilicus | - | - | 6.0 |
| Jun et al.[^40^](https://sciwheel.com/work/citation?ids=11698876&pre=&suf=&sa=0&dbf=0) | 2005 | 68 | M | IV | Body, tail | Adenocarcinoma | Upper extremity, chest | Y | 0.0 | - |
| Otegbayo et al.[^41^](https://sciwheel.com/work/citation?ids=11698975&pre=&suf=&sa=0&dbf=0) | 2005 | 59 | M | IV | - | Adenocarcinoma | Face, chest, abdomen, back | Y | 0.0 | - |
| Tokai et al.[^42^](https://sciwheel.com/work/citation?ids=12096281&pre=&suf=&sa=0&dbf=0) | 2005 | 60 | F | IV | Body | Adenocarcinoma | Umbilicus | Y | 0.0 | 15.0 |
| Inadomi[^43^](https://sciwheel.com/work/citation?ids=11698990&pre=&suf=&sa=0&dbf=0) | 2005 | 82 | M | IV | Body | Adenocarcinoma | Umbilicus | Y | 0.0 | 5.0 |
| Ambro et al.[^44^](https://sciwheel.com/work/citation?ids=11698991&pre=&suf=&sa=0&dbf=0) | 2006 | 63 | M | IV | - | Adenocarcinoma | Scalp | Y | 0.0 | 13.0, alive |
| ^c^Nagato et al.[^45^](https://sciwheel.com/work/citation?ids=12096282&pre=&suf=&sa=0&dbf=0) | 2006 | 73 | F | - | Body | - | Umbilicus | - | - | 6.0 |
| Takemura et al.[^46^](https://sciwheel.com/work/citation?ids=11698996&pre=&suf=&sa=0&dbf=0) | 2007 | 85 | M | IV | Head | Adenocarcinoma | Face | Y | 0.0 | 27.0 |
| Yendluri et al.[^47^](https://sciwheel.com/work/citation?ids=11698997&pre=&suf=&sa=0&dbf=0) | 2007 | 82 | F | IV | Tail | Adenocarcinoma | Umbilicus | Y | 0.0 | - |
| Asai et al.[^48^](https://sciwheel.com/work/citation?ids=11698999&pre=&suf=&sa=0&dbf=0) | 2007 | 79 | F | IV | Body, tail | Adenocarcinoma | Umbilicus | Y | 0.0 | 6.0 |
| Limmathurotsakul et al.[^49^](https://sciwheel.com/work/citation?ids=11699001&pre=&suf=&sa=0&dbf=0) | 2007 | 73 | F | IV | Tail | Adenocarcinoma | Umbilicus | Y | 0.0 | 6.0, alive |
| Hafez et al.[^50^](https://sciwheel.com/work/citation?ids=11698871&pre=&suf=&sa=0&dbf=0) | 2008 | 55 | F | III | Head | Adenocarcinoma | Neck | N | 5.0 | - |
| Ulla et al.[^51^](https://sciwheel.com/work/citation?ids=11699003&pre=&suf=&sa=0&dbf=0) | 2008 | 76 | F | IV | Tail | Adenocarcinoma | Lower extremity | Y | 0.0 | - |
| ^c^Hayami et al.[^52^](https://sciwheel.com/work/citation?ids=11699008&pre=&suf=&sa=0&dbf=0) | 2008 | 84 | F | - | Tail | - | Umbilicus | - | - | 4.0 |
| ^c^Yamashita et al.[^53^](https://sciwheel.com/work/citation?ids=11699011&pre=&suf=&sa=0&dbf=0) | 2008 | 68 | M | - | Body | - | Umbilicus | - | - | 4.0 |
| Colla et al.[^54^](https://sciwheel.com/work/citation?ids=11698873&pre=&suf=&sa=0&dbf=0) | 2009 | 56 | M | IV | Body | Adenocarcinoma | Umbilicus | Y | 0.0 | 0.3 |
| Van Akkooi et al.[^55^](https://sciwheel.com/work/citation?ids=11699012&pre=&suf=&sa=0&dbf=0) | 2010 | 59 | M | IV | - | Adenocarcinoma | Scalp | Y | 0.0 | 4.0 |
| Bdeiri et al.[^56^](https://sciwheel.com/work/citation?ids=11699013&pre=&suf=&sa=0&dbf=0) | 2010 | 70 | F | IV | Tail | Adenocarcinoma | Scalp | Y | 0.0 | 5.0 |
| Pontinen et al. [^57^](https://sciwheel.com/work/citation?ids=11699015&pre=&suf=&sa=0&dbf=0) | 2010 | 73 | F | IV | Tail | Adenocarcinoma | Abdomen | Y | 0.0 | 3.0 |
| Bhat et al.[^58^](https://sciwheel.com/work/citation?ids=11699029&pre=&suf=&sa=0&dbf=0) | 2010 | 59 | F | IV | Tail | Adenocarcinoma | Scalp | Y | 0.0 | - |
| Shimizu et al.[^59^](https://sciwheel.com/work/citation?ids=11699032&pre=&suf=&sa=0&dbf=0) | 2010 | 49 | F | IV | Head | Adenocarcinoma | Lower extremity | Y | 0.0 | 6.0 |
| ^c^Hirahara et al.[^60^](https://sciwheel.com/work/citation?ids=11699035&pre=&suf=&sa=0&dbf=0) | 2010 | 72 | F | - | Tail | - | Umbilicus | - | - | 32.0 |
| Saif et al.[^61^](https://sciwheel.com/work/citation?ids=11699039&pre=&suf=&sa=0&dbf=0) | 2011 | 46 | F | IIB | - | Adenocarcinoma | Chest, abdomen, neck | N | 31.1 | - |
| Ozaki et al.[^62^](https://sciwheel.com/work/citation?ids=11699040&pre=&suf=&sa=0&dbf=0) | 2011 | 70 | F | - | Tail | Adenocarcinoma | Umbilicus | N | 5.0 | 4.0 |
| Ozaki et al.[^62^](https://sciwheel.com/work/citation?ids=11699040&pre=&suf=&sa=0&dbf=0) | 2011 | 81 | M | IV | Body, tail | Adenocarcinoma | Umbilicus | Y | 0.0 | 6.0 |
| Ozaki et al.[^62^](https://sciwheel.com/work/citation?ids=11699040&pre=&suf=&sa=0&dbf=0) | 2011 | 59 | M | - | Body | Adenocarcinoma | Umbilicus | N | 8.0 | 11.0, alive |
| Ozaki et al.[^62^](https://sciwheel.com/work/citation?ids=11699040&pre=&suf=&sa=0&dbf=0) | 2011 | 66 | F | - | Body | - | Umbilicus | Y | 0.0 | 18.0 |
| Horino et al.[^63^](https://sciwheel.com/work/citation?ids=11698892&pre=&suf=&sa=0&dbf=0) | 2012 | 58 | F | - | Body | Adenocarcinoma | Abdomen | - | - | 10.0 |
| Horino et al.[^63^](https://sciwheel.com/work/citation?ids=11698892&pre=&suf=&sa=0&dbf=0) | 2012 | 65 | F | - | Tail | Adenocarcinoma | Abdomen | - | - | 4.0 |
| Kaoutzanis et al.[^64^](https://sciwheel.com/work/citation?ids=11698872&pre=&suf=&sa=0&dbf=0) | 2013 | 43 | M | IV | - | Adenocarcinoma | Scalp | Y | 0.0 | 4.0 |
| Zhou et al.[^65^](https://sciwheel.com/work/citation?ids=10528608&pre=&suf=&sa=0&dbf=0) | 2014 | 76 | F | IV | Tail | Adenocarcinoma | Scalp, chest, abdomen, upper extremity | Y | 0.0 | 2.0 |
| Shin et al.[^66^](https://sciwheel.com/work/citation?ids=11699041&pre=&suf=&sa=0&dbf=0) | 2015 | 60 | M | IV | Body | Neuroendocrine | Lower extremity | Y | 0.0 | 4.0, alive |
| Pandey et al.[^67^](https://sciwheel.com/work/citation?ids=11699042&pre=&suf=&sa=0&dbf=0) | 2016 | 44 | M | IV | Head | Adenocarcinoma | Abdomen | N | 41.0 | 12.0 |
| Kotsantis et al.[^68^](https://sciwheel.com/work/citation?ids=11698883&pre=&suf=&sa=0&dbf=0) | 2017 | 62 | M | - | Head | Adenocarcinoma | Scrotum, abdomen, chest, back, lower extremity | N | 3.4 | - |
| Amirian et al.[^69^](https://sciwheel.com/work/citation?ids=11699043&pre=&suf=&sa=0&dbf=0) | 2017 | 65 | M | IV | Body, tail | Adenocarcinoma | Scrotum | Y | 0.0 | 3.0, alive |
| Laschinger et al.[^70^](https://sciwheel.com/work/citation?ids=11699044&pre=&suf=&sa=0&dbf=0) | 2018 | 67 | F | IV | Body, tail | Neuroendocrine | Lower extremity, breast, back | Y | 0.0 | - |
| Shi et al.[^71^](https://sciwheel.com/work/citation?ids=11698870&pre=&suf=&sa=0&dbf=0) | 2020 | 49 | F | IV | Tail | Adenocarcinoma | Labia | Y | 0.0 | 6.0 |
| Ito et al.[^72^](https://sciwheel.com/work/citation?ids=11699045&pre=&suf=&sa=0&dbf=0) | 2020 | 71 | F | IV | Tail | Adenocarcinoma | Scalp | Y | 0.0 | 10.0, alive |
| Ramachandran et al.[^73^](https://sciwheel.com/work/citation?ids=11698875&pre=&suf=&sa=0&dbf=0) | 2020 | 76 | M | IV | Head | Adenocarcinoma | Scalp | Y | 0.0 | 6.4 |
| Leyrat et al.[^74^](https://sciwheel.com/work/citation?ids=11698888&pre=&suf=&sa=0&dbf=0) | 2021 | 58 | M | IV | Body, tail | Adenocarcinoma | Umbilicus | N | 6.9 | 4.9 |

**Supplementary Table 2. Literature Summary of Pancreas Cancer and Cutaneous Metastases.**

Year refers to publication year of the article. Age refers to age of diagnosis of pancreatic cancer. ^a^Data is presented from table in Shi et al., 2020.[^71^](https://sciwheel.com/work/citation?ids=11698870&pre=&suf=&sa=0&dbf=0) ^b^Data is presented from table in Miyahara et al., 1996.[^27^](https://sciwheel.com/work/citation?ids=11698893&pre=&suf=&sa=0&dbf=0) ^c^Data is presented from table in Horino et al., 2012.[^63^](https://sciwheel.com/work/citation?ids=11698892&pre=&suf=&sa=0&dbf=0) ^d^Cutaneous metastasis site associated with fine-needle tract, drain, or abdominal surgery. *Individual patient data in Lookingbill et al. is not detailed; aggregate data of two patients with cutaneous metastases from pancreatic cancer were provided, with the involved sites including the abdomen, chest, upper extremities, and an average survival of 6 months from diagnosis of cutaneous metastases.

**References for Supplementary Table 2**

[1.    Edelstein JM. Pancreatic carcinoma with unusual metastasis to the skin and subcutaneous tissue simulating cellulitis. *N Engl J Med*. 1950;242(20):779-781. doi:10.1056/NEJM195005182422003](https://sciwheel.com/work/bibliography/11698897)

[2.    Horn JJ, Fred HL, Lane M, Hudgins PT. UMBILICAL METASTASES. *Arch Intern Med*. 1964;114:799-802. doi:10.1001/archinte.1964.03860120111012](https://sciwheel.com/work/bibliography/11698899)

[3.    Barrow MV. Metastatic tumors of the umbilicus. *J Chronic Dis*. 1966;19(10):1113-1117. doi:10.1016/0021-9681(66)90144-5](https://sciwheel.com/work/bibliography/11698900)

[4.    Colin-Jones DG, Copping RM, Gibbs DD, Sharr MM. Malignant Zollinger-Ellison syndrome with gastrin-containing skin metastases. *Lancet*. 1969;1(7593):492-494. doi:10.1016/s0140-6736(69)91591-8](https://sciwheel.com/work/bibliography/11698901)

[5.    Sakai, S. A case of cutaneous metastases from pancreatic carcinoma showing clinical feature of the herpes zoster. *Rinsho Derma (Tokyo)*. Published online 1969.](https://sciwheel.com/work/bibliography/11698903)

[6.    Bordin GM, Weitzner S. Cutaneous metastases as a manifestation of internal carcinoma: diagnostic and prognostic significance. *Am Surg*. 1972;38(11):629-634.](https://sciwheel.com/work/bibliography/11698904)

[7.    Charoenkul V, DelCampo A, Derby A, Hodgson WJ, McElhinney AJ. Tumors of the umbilicus. *Mt Sinai J Med*. 1977;44(2):257-262.](https://sciwheel.com/work/bibliography/11698905)

[8.    Chakraborty AK, Reddy AN, Grosberg SJ, Wapnick S. Pancreatic carcinoma with dissemination to umbilicus and skin. *Arch Dermatol*. 1977;113(6):838-839. doi:10.1001/archderm.1977.01640060134021](https://sciwheel.com/work/bibliography/11698906)

[9.    Weiland FL, Carretta RF. 67Ga-citrate accumulation in a Sister Mary Joseph nodule. *Clin Nucl Med*. 1978;3(8):335-336. doi:10.1097/00003072-197808000-00011](https://sciwheel.com/work/bibliography/11698907)

[10.   Scarpa FJ, Dineen JP, Boltax RS. Visceral neoplasia presenting at the umbilicus. *J Surg Oncol*. 1979;11(4):351-359. doi:10.1002/jso.2930110410](https://sciwheel.com/work/bibliography/11698909)

[11.   Ferrucci JT, Wittenberg J, Margolies MN, Carey RW. Malignant seeding of the tract after thin-needle aspiration biopsy. *Radiology*. 1979;130(2):345-346. doi:10.1148/130.2.345](https://sciwheel.com/work/bibliography/11698911)

[12.   Smith FP, Macdonald JS, Schein PS, Ornitz RD. Cutaneous seeding of pancreatic cancer by skinny-needle aspiration biopsy. *Arch Intern Med*. 1980;140(6):855. doi:10.1001/archinte.1980.00330180129042](https://sciwheel.com/work/bibliography/11698912)

[13.   Chatterjee SN, Bauer HM. Umbilical metastasis from carcinoma of the pancreas. *Arch Dermatol*. 1980;116(8):954-955.](https://sciwheel.com/work/bibliography/11698915)

[14.   Shvili D, Halevy S, Sandbank M. Umbilical metastasis as the presenting sign of pancreatic adenocarcinoma. *Cutis*. 1983;31(5):555-556, 558.](https://sciwheel.com/work/bibliography/11698917)

[15.   Fröhlich E, Frühmorgen P, Seeliger H. [Cutaneous implantation metastasis after fine needle puncture of a pancreatic cancer]. *Ultraschall Med*. 1986;7(3):141-144. doi:10.1055/s-2007-1011933](https://sciwheel.com/work/bibliography/11698918)

[16.   Rashleigh-Belcher HJ, Russell RC, Lees WR. Cutaneous seeding of pancreatic carcinoma by fine-needle aspiration biopsy. *Br J Radiol*. 1986;59(698):182-183. doi:10.1259/0007-1285-59-698-182](https://sciwheel.com/work/bibliography/11698920)

[17.   Hisamoto K, Nishioka K, Ota T, Matsuoka T. A case of umbilical metastasis from carcinoma of the pancreas. *Rinsho Hifuka*. 1987;41:1097–1102.](https://sciwheel.com/work/bibliography/11698926)

[18.   Bergenfeldt M, Genell S, Lindholm K, Ekberg O, Aspelin P. Needle-tract seeding after percutaneous fine-needle biopsy of pancreatic carcinoma. Case report. *Acta Chir Scand*. 1988;154(1):77-79.](https://sciwheel.com/work/bibliography/11698929)

[19.   Sironi M, Radice F, Taccagni GL, Braga M, Zerbi M. Fine needle aspiration of a pancreatic oxyphilic carcinoma with pulmonary and subcutaneous metastases. *Cytopathology*. 1991;2(6):303-309. doi:10.1111/j.1365-2303.1991.tb00505.x](https://sciwheel.com/work/bibliography/11698930)

[20.   Lookingbill DP, Spangler N, Helm KF. Cutaneous metastases in patients with metastatic carcinoma: a retrospective study of 4020 patients. *J Am Acad Dermatol*. 1993;29(2 Pt 1):228-236. doi:10.1016/0190-9622(93)70173-q](https://sciwheel.com/work/bibliography/11698867)

[21.   Siriwardena A, Samarji WN. Cutaneous tumour seeding from a previously undiagnosed pancreatic carcinoma after laparoscopic cholecystectomy. *Ann R Coll Surg Engl*. 1993;75(3):199-200.](https://sciwheel.com/work/bibliography/11698931)

[22.   Taniguchi S, Hisa T, Hamada T. Cutaneous metastases of pancreatic carcinoma with unusual clinical features. *J Am Acad Dermatol*. 1994;31(5 Pt 2):877-880. doi:10.1016/s0190-9622(94)70250-0](https://sciwheel.com/work/bibliography/11698880)

[23.   Ohashi N, lizumi Y, Komatsu T. Two cases with metastatic skin cancer originally from pancreatic carcinoma. *Skin Cancer*. 1995;10:395-399.](https://sciwheel.com/work/bibliography/11698983)

[24.   Fukui Y, Jo N, Maeshima S, Sakatani S, Kusakabe H, Kiyokane K. A Statistical Analysis of Thirty-two Cases of Metastatic Skin Cance. *Hifu*. 1995;37:534-543.](https://sciwheel.com/work/bibliography/12096255)

[25.   Puri AS, Saraswat VA, Krishnani N, Salunke PN. Cutaneous metastases in pancreatic adenocarcinoma. *Indian J Pathol Microbiol*. 1995;38(1):99-101.](https://sciwheel.com/work/bibliography/11698939)

[26.   Nakano S, Narita R, Yamamoto M, Ogami Y, Osuki M. Two cases of pancreatic cancer associated with skin metastases. *Am J Gastroenterol*. 1996;91(2):410-411.](https://sciwheel.com/work/bibliography/11698940)

[27.   Miyahara M, Hamanaka Y, Kawabata A, et al. Cutaneous metastases from pancreatic cancer. *Int J Pancreatol*. 1996;20(2):127-130. doi:10.1007/BF02825511](https://sciwheel.com/work/bibliography/11698893)

[28.   Lesur, G. Peritoneal carcinosis with cutaneous metastases in an endocrine tumor of the pancreas. *Ann Med Intern*. Published online 1997.](https://sciwheel.com/work/bibliography/11698941)

[29.   Horino K, Hiraoka T, Kanemitsu K, et al. Subcutaneous metastases after curative resection for pancreatic carcinoma: a case report and review of the literature. *Pancreas*. 1999;19(4):406-408. doi:10.1097/00006676-199911000-00013](https://sciwheel.com/work/bibliography/11698942)

[30.   Flórez A, Rosón E, Sánchez-Aguilar D, Peteiro C, Toribio J. Solitary cutaneous metastasis on the buttock: a disclosing sign of pancreatic adenocarcinoma. *Clin Exp Dermatol*. 2000;25(3):201-203. doi:10.1046/j.1365-2230.2000.00614.x](https://sciwheel.com/work/bibliography/11698944)

[31.   Kamata A, IIda K. A case of Sister MaryJoseph’s Nodule . *Hifuka No Rinsho*. 2000;42:1406–1407.](https://sciwheel.com/work/bibliography/11698946)

[32.   Gawrieh S, Massey BT, Komorowski RA. Scalp metastases as the first manifestation of pancreatic cancer. *Dig Dis Sci*. 2002;47(7):1469-1471. doi:10.1023/a:1015842413562](https://sciwheel.com/work/bibliography/11698947)

[33.   Takeuchi H, Kawano T, Toda T, et al. Cutaneous metastasis from pancreatic adenocarcinoma: a case report and a review of the literature. *Hepatogastroenterology*. 2003;50(49):275-277.](https://sciwheel.com/work/bibliography/11698948)

[34.   Zhang Y, Selvaggi SM. Metastatic islet cell carcinoma to the umbilicus: diagnosis by fine-needle aspiration. *Diagn Cytopathol*. 2003;29(2):91-94. doi:10.1002/dc.10305](https://sciwheel.com/work/bibliography/11698950)

[35.   Yoneda Y, Tawara J, Takayama Y, Nagahara H, Shiratori K. Two cases of umbilical metastatic tumor from pancreatic cancer. Report of Sister Mary Joseph’s nodule. *J Jpn Panc Soc*. 2003;18:507-511.](https://sciwheel.com/work/bibliography/12096270)

[36.   Fiori E, Galati G, Bononi M, et al. Subcutaneous metastasis of pancreatic cancer in the site of percutaneous biliary drainage. *J Exp Clin Cancer Res*. 2003;22(1):151-154.](https://sciwheel.com/work/bibliography/11698971)

[37.   St Peter SD, Nguyen CC, Mulligan DC, Moss AA. Subcutaneous metastasis at a surgical drain site after the resection of pancreatic cancer. *Int J Gastrointest Cancer*. 2003;33(2-3):111-115. doi:10.1385/ijgc:33:2-3:111](https://sciwheel.com/work/bibliography/11698972)

[38.   Crescentini F, Deutsch F, Sobrado CW, Araújo S de. Umbilical mass as the sole presenting symptom of pancreatic cancer: a case report. *Rev Hosp Clin Fac Med Sao Paulo*. 2004;59(4):198-202. doi:10.1590/s0041-87812004000400008](https://sciwheel.com/work/bibliography/11698973)

[39.   Okazaki M, Hiratsuka M, Okuno S. A case of the pancreas body cancer finded out by a Sister Mary Joseph’s Nodule. *Tann To Sui*. 2004;25:451-453.](https://sciwheel.com/work/bibliography/12096273)

[40.   Jun DW, Lee OY, Park CK, et al. Cutaneous metastases of pancreatic carcinoma as a first clinical manifestation. *Korean J Intern Med*. 2005;20(3):260-263. doi:10.3904/kjim.2005.20.3.260](https://sciwheel.com/work/bibliography/11698876)

[41.   Otegbayo JA, Oluwasola OA, Akere A, Yakubu A, Daramola OOM, Ogun GO. Pancreatic carcinoma presenting as cutaneous nodules in a diabetic Nigerian male. *West Afr J Med*. 2005;24(2):180.](https://sciwheel.com/work/bibliography/11698975)

[42.   Tokai H, Matsuo S, Azum T, Haraguchi M, Yamaguchi S, Kanematsu T. Pancreatic cancer with umbilical metastases (Sister Mary Joseph’s Nodule). *Acta Medica*. 2005;50(3):123-126.](https://sciwheel.com/work/bibliography/12096281)

[43.   Inadomi T. Sister Mary Joseph’s nodule: a clue to finding pancreatic cancer in a patient previously affected by gastric cancer. *Eur J Dermatol*. 2005;15(6):492-494.](https://sciwheel.com/work/bibliography/11698990)

[44.   Ambro CM, Humphreys TR, Lee JB. Epidermotropically metastatic pancreatic adenocarcinoma. *Am J Dermatopathol*. 2006;28(1):60-62. doi:10.1097/01.dad.0000157460.72334.fa](https://sciwheel.com/work/bibliography/11698991)

[45.   Nagato M, Manabe M, Umebayashi Y. A case of Sister Mary Joseph’s Nodule derived from pancreatic cancer. *Hifuka No Rinsho*. 2006;48:479-480.](https://sciwheel.com/work/bibliography/12096282)

[46.   Takemura N, Fujii N, Tanaka T. Cutaneous metastasis as the first clinical manifestation of pancreatic adenocarcinoma: a case treated with gemcitabine. *J Dermatol*. 2007;34(9):662-664. doi:10.1111/j.1346-8138.2007.00353.x](https://sciwheel.com/work/bibliography/11698996)

[47.   Yendluri V, Centeno B, Springett GM. Pancreatic cancer presenting as a Sister Mary Joseph’s nodule: case report and update of the literature. *Pancreas*. 2007;34(1):161-164. doi:10.1097/01.mpa.0000240602.18688.43](https://sciwheel.com/work/bibliography/11698997)

[48.   Asai K, Hiramitsu Y, Yoneda K, Nakura K, Yamada T, Yoshida M. A case of Sister Mary Yoseph’s Nodule. *Skin Cancer*. 2007;22:136–139.](https://sciwheel.com/work/bibliography/11698999)

[49.   Limmathurotsakul D, Rerknimitr P, Korkij W, Noppakun N, Kullavanijaya P, Rerknimitr R. Metastatic mucinous cystic adenocarcinoma of the pancreas presenting as Sister Mary Joseph’s nodule. *JOP*. 2007;8(3):344-349.](https://sciwheel.com/work/bibliography/11699001)

[50.   Hafez HZA. Cutaneous pancreatic metastasis: a case report and review of literature. *Indian J Dermatol*. 2008;53(4):206-209. doi:10.4103/0019-5154.44806](https://sciwheel.com/work/bibliography/11698871)

[51.   Ulla JL, Garcia-Doval I, Posada C, et al. Plantar keratoderma as a presenting sign of pancreatic adenocarcinoma. *J Clin Ultrasound*. 2008;36(2):108-109. doi:10.1002/jcu.20377](https://sciwheel.com/work/bibliography/11699003)

[52.   Hayami M, Wakai T, Kaneko K, Maruyama T, Shirai Y, Hatayama K. A case of pancreas cancer derived from Sister Mary Yoseph’s Nodule . *Niigata Igakkai Zassi*. 2008;122:148-152.](https://sciwheel.com/work/bibliography/11699008)

[53.   Yamashita S, Sakon M, Hiura Y, et al. [A case of metastases of umbilicus (Sister Mary Joseph’s nodule)]. *Gan To Kagaku Ryoho*. 2008;35(12):2112-2114.](https://sciwheel.com/work/bibliography/11699011)

[54.   Colla TG, Lovatto L, Duquia RP. Case for diagnosis: umbilical metastasis of pancreatic carcinoma (Sister Mary Joseph’s Nodule). *An Bras Dermatol*. 2009;84(3):297-298. doi:10.1590/s0365-05962009000300015](https://sciwheel.com/work/bibliography/11698873)

[55.   van Akkooi ACJ, Dokter J, Boxma H. Unusual first presentation of metastatic pancreatic cancer as skin metastases in a burn patient. *Burns*. 2010;36(6):e111-4. doi:10.1016/j.burns.2009.12.004](https://sciwheel.com/work/bibliography/11699012)

[56.   Bdeiri K, Kamar FG. Cutaneous metastasis of pancreatic adenocarcinoma as a first clinical manifestation: a case report and review of the literature. *Gastrointest Cancer Res*. 2013;6(2):61-63.](https://sciwheel.com/work/bibliography/11699013)

[57.   Pontinen T, Melin A, Varadi G, et al. Cutaneous metastasis of pancreatic adenocarcinoma after kidney transplant: a case report and review of the literature. *Exp Clin Transplant*. 2010;8(4):273-276.](https://sciwheel.com/work/bibliography/11699015)

[58.   Bhat W, Abood A, Maraveyas A. Cutaneous metastasis from pancreatic carcinoma: a case report and review. *J Clin Exp Dermatol Res*. 2010;1:206-111.](https://sciwheel.com/work/bibliography/11699029)

[59.   Shimizu H, Maegawa J, Ho T, Yamamoto Y, Mikami T, Nagahama K. Cutaneous metastasis of pancreatic carcinoma as an initial symptom in the lower extremity with obstructive lymphedema treated by physiotherapy and lymphaticovenous shunt: a case report, review, and pathophysiological implications. *Lymphology*. 2010;43(1):19-24.](https://sciwheel.com/work/bibliography/11699032)

[60.   Hirahara N, Nisi T, Kawabata Y, et al. A case of pancreas tail cancer lived for 20 months after chemotherapy by Gemcitabine . *Kan Tan Sui*. 2010;60:725-730.](https://sciwheel.com/work/bibliography/11699035)

[61.   Saif MW, Brennan M, Penney R, Hotchkiss S, Kaley K. Cutaneous metastasis in a patient with pancreatic cancer. *JOP*. 2011;12(3):306-308.](https://sciwheel.com/work/bibliography/11699039)

[62.   Ozaki N, Takamori H, Baba H. Sister Mary Joseph’s nodule derived from pancreatic cancer. *J Hepatobiliary Pancreat Sci*. 2011;18(1):119-121. doi:10.1007/s00534-010-0296-y](https://sciwheel.com/work/bibliography/11699040)

[63.   Horino K, Takamori H, Ikuta Y, et al. Cutaneous metastases secondary to pancreatic cancer. *World J Gastrointest Oncol*. 2012;4(7):176-180. doi:10.4251/wjgo.v4.i7.176](https://sciwheel.com/work/bibliography/11698892)

[64.   Kaoutzanis C, Chang MC, Abdul Khalek FJ, Kreske E. Non-umbilical cutaneous metastasis of a pancreatic adenocarcinoma. *BMJ Case Rep*. 2013;2013. doi:10.1136/bcr-2012-007931](https://sciwheel.com/work/bibliography/11698872)

[65.   Zhou H-Y, Wang X-B, Gao F, Bu B, Zhang S, Wang Z. Cutaneous metastasis from pancreatic cancer: A case report and systematic review of the literature. *Oncol Lett*. 2014;8(6):2654-2660. doi:10.3892/ol.2014.2610](https://sciwheel.com/work/bibliography/10528608)

[66.   Shin WY, Lee KY, Ahn SI, Park S-Y, Park K-M. Cutaneous metastasis as an initial presentation of a non-functioning pancreatic neuroendocrine tumor. *World J Gastroenterol*. 2015;21(33):9822-9826. doi:10.3748/wjg.v21.i33.9822](https://sciwheel.com/work/bibliography/11699041)

[67.   Pandey P, Al-Rohil RN, Goldstein JB, et al. Cutaneous metastasis of a mucoepidermoid carcinoma of the pancreas: first reported case. *Am J Dermatopathol*. 2016;38(11):852-856. doi:10.1097/DAD.0000000000000604](https://sciwheel.com/work/bibliography/11699042)

[68.   Kotsantis I, Economopoulou P, Dritsakos K, et al. Extensive cutaneous metastases of pancreatic adenocarcinoma: a case report and review of the literature. *Clin Case Rep*. 2017;5(1):51-56. doi:10.1002/ccr3.737](https://sciwheel.com/work/bibliography/11698883)

[69.   Amirian MJ, Arnouk AM, Healy KA. Metastatic pancreatic adenocarcinoma to the scrotum. *Turk J Urol*. 2017;43(2):220-222. doi:10.5152/tud.2017.58235](https://sciwheel.com/work/bibliography/11699043)

[70.   Laschinger ME, Naga L, Gaspari AA. Cutaneous metastases of pancreatic neuroendocrine carcinoma. *G Ital Dermatol Venereol*. 2018;153(5):722-724. doi:10.23736/S0392-0488.17.05103-3](https://sciwheel.com/work/bibliography/11699044)

[71.   Shi Y, Li S-S, Liu D-Y, Yu Y. Cutaneous metastases of pancreatic carcinoma to the labia majora: A case report and review of literature. *World J Gastrointest Oncol*. 2020;12(11):1372-1380. doi:10.4251/wjgo.v12.i11.1372](https://sciwheel.com/work/bibliography/11698870)

[72.   Ito H, Tajiri T, Hiraiwa S-I, et al. A Case of Rare Cutaneous Metastasis from Advanced Pancreatic Cancer. *Case Rep Oncol*. 2020;13(1):49-54. doi:10.1159/000505322](https://sciwheel.com/work/bibliography/11699045)

[73.   Ramachandran P, Boyapati L, Joseph G. Scalp metastasis as the first presentation of an underlying aggressive pancreatic cancer. *J Investig Med High Impact Case Rep*. 2020;8:2324709620931667. doi:10.1177/2324709620931667](https://sciwheel.com/work/bibliography/11698875)

[74.   Leyrat B, Bernadach M, Ginzac A, Lusho S, Durando X. Sister Mary Joseph Nodules: A Case Report about a Rare Location of Skin Metastasis. *Case Rep Oncol*. 2021;14(1):664-670. doi:10.1159/000515298](https://sciwheel.com/work/bibliography/11698888)
